# DDB2 expression lights the way for precision radiotherapy response in PDAC cells, with or without olaparib

**DOI:** 10.1038/s41420-024-02188-9

**Published:** 2024-09-27

**Authors:** Julie Dardare, Andréa Witz, Margaux Betz, Aurélie François, Laureline Lamy, Marie Husson, Jessica Demange, Marie Rouyer, Aurélien Lambert, Jean-Louis Merlin, Pauline Gilson, Alexandre Harlé

**Affiliations:** 1 Université de Lorraine, Centre National de la Recherche Scientifique (CNRS), Unité Mixte de Recherche (UMR) 7039 Centre de Recherche en Automatique de Nancy (CRAN), Nancy, France; 2 Service de Biopathologie, Institut de Cancérologie de Lorraine, Vandœuvre-lès-Nancy, France; 3 Département d’oncologie médicale, Institut de Cancérologie de Lorraine, Vandœuvre-lès-Nancy, France

**Keywords:** Cancer, Radiotherapy

## Abstract

Pancreatic ductal adenocarcinoma (PDAC) is one of the deadliest cancers. Therapeutic options for PDAC are primarily restricted to surgery in the early stages of the disease or chemotherapy in advanced disease. Only a subset of patients with germline defects in *BRCA1/2* genes can potentially benefit from personalized therapy, with the PARP inhibitor olaparib serving as a maintenance treatment for metastatic disease. Although the role of radiotherapy in PDAC remains controversial, the use of radiosensitizers offers hope for improving cancer management. Previously, we have shown that damage-specific DNA binding protein 2 (DDB2) is a potential prognostic and predictive biomarker for chemotherapy response in PDAC. In this study, we investigated the function of DDB2 in radiotherapy response, with and without radiosensitization by olaparib in PDAC cells. Our findings demonstrated DDB2 resistance to radiation effects, thereby improving cell survival and enhancing the repair of ionizing radiation-induced DNA double-strand breaks. We observed that DDB2 expression enhances the cell cycle arrest in the G2 phase by phosphorylating Chk1 and Chk2 cell cycle checkpoints. Additionally, we identified a novel link between DDB2 and PARP1 in the context of radiotherapy, which enhances the expression and activity of PARP1. Our findings highlight the potential of low-DDB2 expression to potentiate the radiosensitization effect of olaparib in PDAC cells. Collectively, this study provides novel insights into the impacts of DDB2 in the radiotherapy response in PDAC, enabling its employment as a potential biomarker to predict resistance to radiation. Furthermore, DDB2 represents a significant step forward in precision radiotherapy by widening the scope of patients who can be benefiting from olaparib as a radiosensitizer. Hence, this research has the potential to enrich the limited use of radiotherapy in the care of patients with PDAC.

## Introduction

Pancreatic ductal adenocarcinoma (PDAC) continues to be one of the most aggressive diseases, with the lowest 5-year relative survival rates estimated at ~12% [1]. Surgery remains the only potentially curative option for 20% of patients diagnosed at an early stage [2]. Chemotherapy plays a pivotal role in the management of patients with locally advanced disease or distant metastases. Although progress has been made with the approval of olaparib, a poly(ADP-ribose) polymerase inhibitor (PARPi), in metastatic PDAC patients with germline mutations in *BRCA1/2* genes, only a small subset of patients can benefit from this precision therapy [3]. The interest in radiotherapy (RT) and chemoradiotherapy (CRT) in PDAC remains controversial. Indeed, the survival benefits of RT alone may be outweighed by treatment-induced toxicity [4]. The use of CRT as adjuvant therapy after resection led to opposite conclusions in clinical trials, being beneficial to patients in some cases or deleterious in other studies [5–7]. However, the use of CRT appears to demonstrate its value as neoadjuvant therapy. Recently, long-term results from the PREOPANC trial, which used neoadjuvant gemcitabine-based CRT, demonstrated improved overall survival (OS) in patients with resectable or borderline resectable pancreatic cancer [8]. Currently, RT is not a standard of care for patients with PDAC, but its effects could be potentiated by selecting RT-sensitive patients, or by using radiosensitizers to provide real benefits to patients.

Ionizing radiation (IR) triggers cell death by inducing DNA damage, primarily through the induction of double-strand breaks (DSB) or single-strand breaks (SSB). A variety of DNA repair mechanisms can act on these damages, including homologous recombination (HR) and non-homologous end-joining (NHEJ). The radiosensitivity of a cell is determined by its ability to repair highly lethal DNA DSB. Consequently, the combination of RT and the inhibition of DNA repair pathways represents a promising avenue for improving radiosensitivity in pancreatic cancer [9]. Among the various proteins involved in DNA repair, the inhibition of the poly(ADP-ribose) polymerase-1 (PARP1) in association with RT has been extensively studied and has demonstrated a promising radiosensitizing effect. For instance, the PARPi olaparib, which has been approved for the treatment of patients with metastatic PDAC since 2019, has demonstrated a radiosensitizing effect on pancreatic cancer cells in vitro. However, the effect was more nuanced in vivo [9–11]. A phase I study has evaluated the association of the PARPi veliparib with gemcitabine-based CRT for locally advanced pancreatic cancer. The treatment was well tolerated by patients, and the authors observed encouraging results [12].

Damage-specific DNA binding protein 2 (DDB2) is a protein that was originally implicated in the recognition of ultraviolet-induced DNA damage and in the initiation of the nucleotide excision repair (NER) pathway [13]. Beyond its well-documented roles in DNA repair, DDB2 has been shown to possess both anti-oncogenic and pro-oncogenic properties, depending on the cancer location [14]. In ovarian [15] and prostate cancers [16], DDB2 has demonstrated antiproliferative activities, whereas it has been observed to promote breast cancer cell proliferation [17]. In addition, DDB2 has been shown to repress epithelial-to-mesenchymal transition (EMT) in colon cancer [18] and in oral/head and neck squamous cell carcinoma (HNSCC) [19]. Furthermore, DDB2 has been demonstrated to reduce breast cancer cell motility and invasiveness [20], while DDB2 has been found to promote migration and invasion of gastric cancer cells [21]. Additionally, DDB2 was identified as being induced in a radioresistant non-small cell lung cancer (NSCLC) cell line [22]. In NSCLC cell lines, DDB2 expression has been demonstrated to promote resistance to radiation by facilitating Chk1 activation upon IR and promoting HR repair [23].

In our previous study, we identified novel tumor suppressor functions of DDB2 in PDAC cell lines. DDB2 appears to inhibit EMT, migration, and invasion. Furthermore, our findings demonstrated that DDB2 expression sensitized cells to 5-fluorouracil (5-FU), oxaliplatin, and gemcitabine chemotherapies by downregulating Bcl-2 expression levels [24]. The objective of this study was to investigate the influence of DDB2 protein expression on the potentiation of RT effects with or without PARPi olaparib in patients with PDAC.

## Results

### Determination of HR status

The PDAC cell models utilized in this study have been previously established and described in detail [24]. In brief, the expression level of DDB2 was decreased in the T3M4 DDB2-low cells by transfection with a DDB2 CRISPR/Cas9 KO plasmid, while the expression level of DDB2 was increased in the Capan-2 DDB2-high cells by transfection with a DDB2 CRISPR activation plasmid. The cellular models are compared to control cells, T3M4 CTRL cells and Capan-2 CTRL cells, respectively, which have been transfected with a control CRISPR plasmid. To assess the capacity of cells to repair their DNA, an analysis of HR status was conducted using NGS. The genomic instability index obtained was negative (Supplementary Table I), and no significant alteration was identified in the 28 analyzed genes for all cell models, consistent with a homologous recombination proficiency status (HRP).

### DDB2 expression favors resistance to radiation in PDAC cell models

**Fig. 1:**
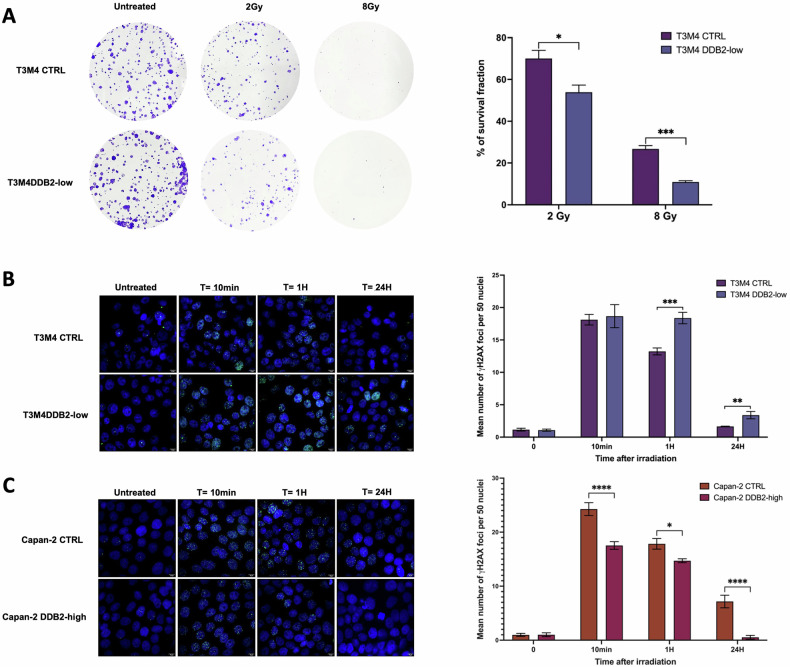
DDB2 induces resistance to ionizing radiations in PDAC cells. **A** The sensitivity of T3M4 CTRL and T3M4 DDB2-low cells to ionizing radiation was determined by clonogenic formation assay. The survival fraction was determined in relation to the number of colonies obtained in untreated cells. The data from three independent experiments are expressed as the mean ± SEM, *p < 0.05 and ***p < 0.001 (Student’s unpaired *t*-test). **B**, **C** The induction of double-strand breaks was analyzed by γ-H2AX foci labeling. The nucleus was counterstained with DAPI (x40). The mean number of γ-H2AX foci in 50 nuclei was determined at 10 min, 1 h, and 24 h after 2 Gy irradiation. The data from three independent experiments are expressed as the mean ± SEM, *p < 0.05, **p < 0.01, ***p < 0.001, and ****p < 0.0001 (ANOVA).

Cell survival of T3M4 cell models after irradiation was determined by clonogenic formation assay. The clonogenic formation assay could not be performed on Capan-2 cells due to their inability to form colonies. The downregulation of DDB2 (T3M4 DDB2-low) resulted in a sensitization of cells to IR, accompanied by a reduction in clonogenic formation. The survival fraction of T3M4 DDB2-low and T3M4 CTRL cells was 53% and 70%, respectively (*p* = 0.0179), after 2 Gy IR, and 10% versus 26% (*p* = 0.0008) after 8 Gy IR (Fig. 1A). The induction of DNA DSB was investigated following exposure to 2 Gy of IR using γ-H2AX foci labeling. The number of foci after irradiation was significantly increased in T3M4 DDB2-low cells at 1 h and 24 h compared to T3M4 CTRL cells (*p* = 0.0075 and *p* = 0.0054, respectively) (Fig. 1B). Conversely, Capan-2 DDB2-high cells exhibited a significant decrease in the number of γ-H2AX foci at 10 min, 1 h, and 24 h compared to Capan-2 CTRL cells (*p* = 0.0041; *p* = 0.0398 and *p* = 0.0056, respectively) (Fig. 1C). At 24 h, residual γ-H2AX foci are indicative of DNA DSB that are generally irreversible and result in cell death. T3M4 CTRL cells demonstrated their capacity to resist IR, with an average of only one persistent γ-H2AX focus per nucleus. In contrast, T3M4 DDB2-low cells exhibited their sensitivity to IR, with an average of four persistent γ-H2AX foci per nucleus. Conversely, Capan-2 CTRL cells demonstrated sensitivity to IR with an average of 7 persistent γ-H2AX foci. In contrast, Capan-2 DDB2-high cells exhibited a near absence of γ-H2AX foci after 24 h of exposure to IR, indicating that the DSB have been repaired and that these cells were likely resistant to IR.

### DDB2 expression increases G2/M arrest upon IR treatment through the regulation of Chk1 and Chk2 expression and phosphorylation

**Fig. 2:**
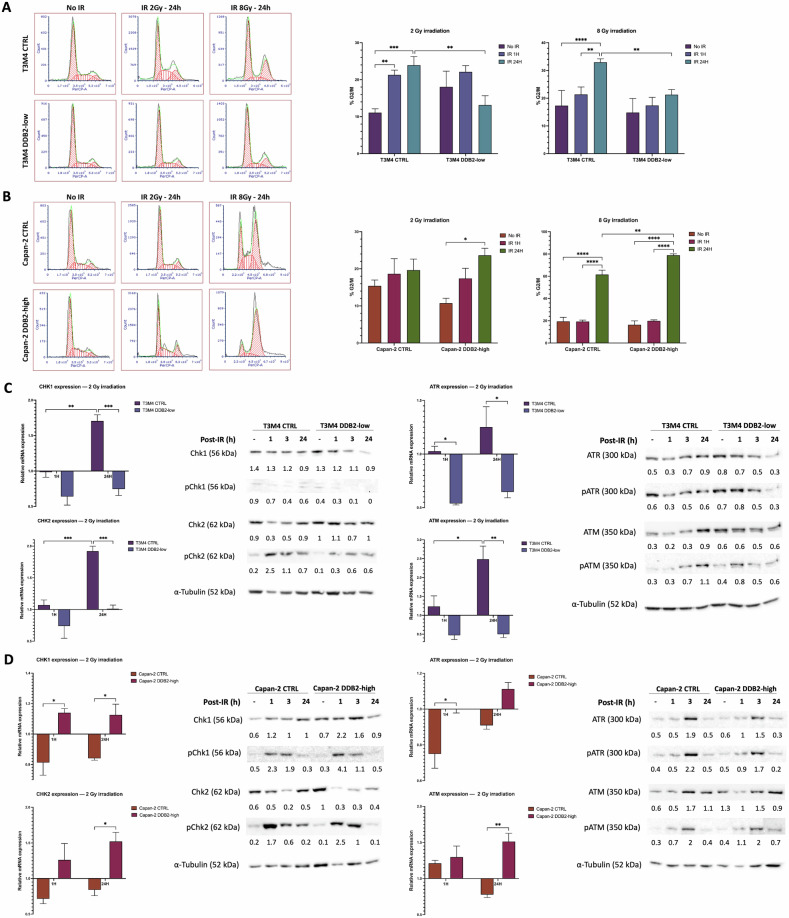
DDB2 affects G2/M arrest through increased expression and phosphorylation of cell cycle checkpoints. The cell cycle distribution was determined following exposure to 2 Gy and 8 Gy ionizing irradiation (IR) in T3M4 cells (**A**) and Capan-2 cells (**B**). The data from three independent experiments are expressed as the mean ± SEM, *p < 0.05, **p < 0.01, ***p < 0.001, and ****p < 0.0001 (ANOVA). The transcriptional and protein expression levels of the cell cycle checkpoint proteins Chk1 and Chk2 and their upstream effectors ATR and ATM were analyzed for T3M4 cells (**C**) and Capan-2 cells (**D**). The transcriptional level expression following 1 h and 24 h of exposure to 2 Gy IR were determined by RT-qPCR. β-actin was used as a housekeeping gene. The results were normalized by the expression of the genes of interest in untreated cells. The data from three independent experiments are expressed as mean ± SEM, *p < 0.05, **p < 0.01, and ***p < 0.001 (ANOVA). Protein expression and phosphorylation were studied by western blot analysis following 1 h, 3 h, and 24 h exposure to 2 Gy IR. α-tubulin was used as a loading control.

A cell cycle phase distribution analysis was conducted at both 1 h and 24 h after 2 Gy and 8 Gy IR treatment. In T3M4 CTRL cells, IR induced a significant G2/M arrest at 1 h (*p* = 0.064) and 24 h (*p* = 0.0008) after 2 Gy treatment, as well as at 24 h after 8 Gy treatment (*p* < 0.0001). The G2/M arrest was abrogated in T3M4 DDB2-low cells following 2 Gy or 8 Gy IR treatment. The proportion of cells in the G2/M phase at 24 h after 2 Gy and 8 Gy IR was significantly increased by ~10% in T3M4 CTRL cells compared to T3M4 DDB2-low cells (*p* = 0.0040 and *p* = 0.0022, respectively) (Fig. 2A). In the Capan-2 CTRL, 2 Gy IR did not induce a significant G2/M arrest, while 8 Gy IR induced G2/M arrest 24 h after irradiation (*p* < 0.0001). Capan-2 DDB2-high cells exhibited a significant G2/M arrest 24 h after 2 Gy and 8 Gy IR (*p* = 0.0397 and *p* < 0.0001, respectively). G2/M arrest was significantly increased by ~17% in Capan-2 DDB2-high cells in comparison to Capan-2 CTRL cells at 24 h following 8 Gy IR (*p* = 0.0078) (Fig. 2B).

Given the established role of Chk2/ATM in G2 arrest upon IR, and the implication of Chk1/ATR in maintaining this arrest, we sought to assess the expression level of these proteins following exposure to 2 Gy IR. First, their expression at the transcriptional level was analyzed by RT-qPCR at 1 h and 24 h after IR. T3M4 CTRL cells exhibited a significant overexpression of Chk2 and ATM (*p* = 0.0007 and *p* = 0.0015, respectively) and a significant increase in Chk1 and ATR expression (*p* = 0.0003 and *p* = 0.0127, respectively) 24 h after IR. In contrast, the transcriptional expression level of the latter was unaltered following IR exposure in T3M4 DDB2-low cells (Fig. 2C). The transcriptional expression levels of Chk2, ATM, and Chk1 were unaltered in Capan-2 CTRL cells. Conversely, Capan-2 DDB2-high cells exhibited a significant overexpression of Chk2 and ATM (*p* = 0.0169 and *p* = 0.0038, respectively) 24 h after IR exposure, accompanied by a significant increase in Chk1 expression at 1 h and 24 h after IR (*p* = 0.0109 and *p* = 0.0383, respectively). Finally, ATR expression remained unaltered in Capan-2 DDB2-high cells, whereas a significant decrease was observed in Capan-2 CTRL cells (*p* = 0.0223) (Fig. 2D).

Subsequently, protein expression levels and their activation by phosphorylation were analyzed at 1 h, 3 h, and 24 h following IR exposure. For each cell model, Chk1 and Chk2 protein expression was maximal in the untreated condition and decreased chronologically after IR, coinciding with the appearance of the phosphorylated proteins pChk1 and pChk2. The level of pChk1 protein expression was very low in T3M4 CTRL cells and even lower in T3M4 DDB2-low cells. However, the level of pChk2 protein expression increased in T3M4 CTRL cells with a protein ratio of 2.5 one hour after IR, while pChk2 expression was very low for T3M4 DDB2-low cells with a protein ratio of 0.3 one hour after IR (Fig. 2C). The expression levels of pChk1 and pChk2 proteins were found to be higher in Capan-2 DDB2-high cells 1 h after IR than in Capan-2 CTRL cells, the protein ratios were 4.1 and 2.5 versus 2.3 and 1.7, respectively (Fig. 2D). The expression levels of ATR and ATM and their phosphorylated forms, pATR and pATM, exhibited a slight increase following irradiation in T3M4 CTRL cells, whereas a decrease was observed in T3M4 DDB2-low cells (Fig. 2C). For Capan-2 CTRL and Capan-2 DDB2-high cells, the expression of these proteins was maximal 3 h after IR, with similar protein ratios (Fig. 2D). These data bring to light the involvement of DDB2 in the regulation of Chk1 and Chk2, suggesting that DDB2 may facilitate their activation following IR exposure.

### DDB2 modulates the radiosensitizing effects of olaparib

**Fig. 3:**
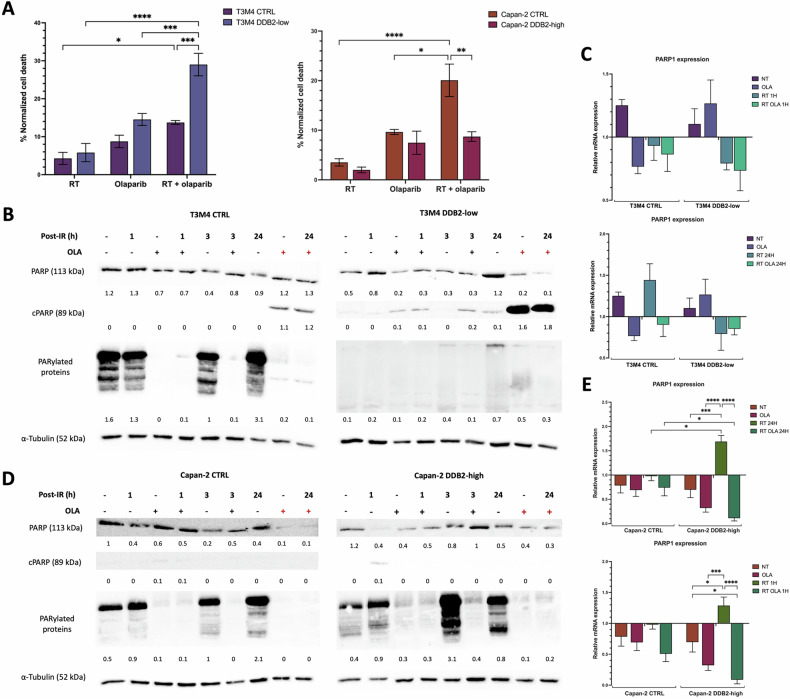
DDB2 impairs the radiosensitizing olaparib effect via PARP1 activity. **A** Cell death was monitored by flow cytometry with annexin V and propidium iodide labeling 24 h after 2 Gy radiotherapy alone or 2 Gy radiotherapy with olaparib pretreatment. **B**, **D** PARP, cleaved PARP (cPARP), and PARylated proteins expression were studied by western blot after 1 h, 3 h, and 24 h exposure to 2 Gy IR with or without olaparib pretreatment. Olaparib was administered for either 24 h (black +) or 48 h (red +) before. α-tubulin was used as a loading control. **C**, **E** PARP1 expression was analyzed by RT-qPCR in different conditions: no treatment (NT), after olaparib treatment (OLA), after radiotherapy treatment (RT), or after radiotherapy with olaparib pretreatment (RT OLA). PARP1 expression was analyzed at 1 h and 24 h post-irradiation. β-actin was used as a housekeeping gene. The expression of untreated cells was normalized to the expression of the corresponding wild-type cell lines, and the expression of the different treatment conditions was normalized to the expression of the corresponding untreated cells. The data from three independent experiments are expressed as the mean ± SEM, *p < 0.05, ***p < 0.001, ****p < 0.0001 (ANOVA).

The inhibitory concentration 50 (IC50) of olaparib in our models was determined using a crystal violet assay. The IC50 values obtained for Capan-2 cells were approximately three times higher than those obtained for T3M4 cells. No significant difference in IC50 was observed between T3M4 CTRL and T3M4 DDB2-low cells, and between Capan-2 CTRL and Capan-2 DDB2-high cells (Table 1), indicating that a change in DDB2 expression is not sufficient to affect olaparib sensitivity in our models. The induction of cell death by RT, olaparib, or a combination of both was analyzed by flow cytometry. The olaparib concentration selected for subsequent experiments was slightly below the IC50 for each cell line: 15 and 50 µM for T3M4 and Capan-2 cells, respectively. Olaparib was administered 24 h prior to the administration of 2 Gy IR. No significant difference was observed between RT and olaparib monotherapy in our cell models. Nevertheless, the combination of therapies resulted in a significant increase in cell death in T3M4 DDB2-low cells compared with RT or olaparib alone (*p* < 0.0001 and *p* = 0.0006, respectively). Cell death was also increased with both therapies in T3M4 CTRL cells in comparison to RT alone (*p* = 0.0447). However, the potentiation of treatments was more pronounced in T3M4 DDB2-low cells, with an average of 29% versus 14% for T3M4 CTRL cells (*p* = 0.0003) (Fig. 3A). These results were corroborated by western blotting, which demonstrated a higher induction of apoptosis associated with increased cleaved PARP (cPARP) in T3M4 DDB2-low cells following 24 h with the treatment combination (Fig. 3B).

**Table 1:** Calculated IC50 of olaparib for T3M4 CTRL, T3M4 DDB2-low, Capan-2 CTRL, and Capan-2 DDB2-high cells.

	T3M4 CTRL	T3M4 DDB2-low	Capan-2 CTRL	Capan-2 DDB2-high
IC50 (µM)	16.35 ± 0.35	16.50 ± 0.51	52.92 ± 0.40	55.24 ± 2.45
*p*	NS		NS

The combination of therapies did not affect cell death in Capan-2 DDB2-high cells, while it increased cell death in Capan-2 CTRL cells compared with RT or olaparib monotherapy (*p* < 0.0001 and *p* = 0.0194, respectively). The combination of treatments induced a greater degree of cell death in Capan-2 DDB2-high cells (9%) than in Capan-2 CTRL cells (20%) (*p* = 0.0041) (Fig. 3A). However, there was no evidence of cPARP induction in Capan-2 CTRL or Capan-2 DDB2-high cells (Fig. 3C). These findings indicate that DDB2 expression can modify the radiosensitizing effect of olaparib in PDAC cells.

### DDB2 affects PARP1 activity

The expression of PARP1, the main target of olaparib, was evaluated following monotherapy with olaparib, RT alone, or olaparib pretreatment in combination with RT. At the transcriptional level, PARP1 expression was not found to be altered in relation to DDB2 expression, either at the basal level or following olaparib treatment. No significant difference was observed in PARP1 expression following RT alone or with olaparib pretreatment for T3M4 CTRL and T3M4 DDB2-low cells. However, PARP1 expression was increased in Capan-2 DDB2-high cells 1 h and 24 h after RT alone for Capan-2 DDB2-high (*p* = 0.0486 and *p* = 0.0009, respectively). This overexpression was significantly reversed by the olaparib pretreatment in Capan-2 DDB2-high cells. In addition, the level of PARP1 was significantly lower in this condition compared to Capan-2 CTRL cells (*p* = 0.0474) (Fig. 3E). At the protein level, PARP1 expression was found to be lower in T3M4 DDB2-low cells than in T3M4 CTRL cells. PARP1 protein levels were notably low in T3M4 DDB2-low cells 48 h after olaparib and 24 h after RT and olaparib pretreatment, which was associated with an increase in cPARP levels. The level of PARP1 activity was quantified by analyzing PARylated proteins. The expression levels of PARylated proteins in paired cell lines are presented in Supplementary Fig. 1. In T3M4 CTRL cells, the level of PARylated proteins was highest 24 h after RT alone and was drastically reduced with olaparib. In T3M4 DDB2-low cells, PARylated protein levels were very low at the basal level and in all treatment conditions. However, there was a slight increase in levels 24 h after RT alone (Fig. 3B). In contrast, Capan-2 DDB2-high cells exhibited comparable PARP1 protein expression than Capan-2 CTRL cells, except 48 h after olaparib and 24 h after RT with olaparib pretreatment, when levels were slightly elevated. The level of PARylated proteins were found to be the highest in Capan-2 DDB2-high cells, with maximal PARP1 activity occurring 3 h after RT alone. In contrast, the maximal PARP1 activity was observed 24 h after RT alone in Capan-2 CTRL cells. These results demonstrated that DDB2 expression was associated with an increase in PARP1 expression and activity (Fig. 3C).

## Discussion

The IR-resistant effect of DDB2 was demonstrated by increased cell survival and more pronounced induction and persistence of γH2AX foci after IR when DDB2 is overexpressed. The number of γH2AX foci induced correlates quantitatively with the number of DSB induced by IR [25]. Once DSB have been repaired, γH2AX foci are removed, and residual foci present 24 hours following IR can be used to determine radiosensitivity [26]. Our findings demonstrate that the number of induced and residual γH2AX foci was higher in cells with low DDB2 levels (T3M4 DDB2-low and Capan-2 CTRL), thereby attesting to their radiosensitivity. Conversely, γH2AX foci were decreased in cells with elevated DDB2 levels (T3M4 CTRL and Capan-2 DDB2-high), indicating their resistance to IR. These observations led to the identification of a link between DDB2 and DSB repair. The repair of IR-induced DSB is primarily accomplished through the HR or NHEJ pathways, both of which are activated during the G2/M cell cycle transition [27, 28]. The cell cycle can be arrested at the G2/M phase in two ways: either by immediate arrest in G2, or by a subsequent accumulation in the G2 phase [29]. In our cell models, the expression of DDB2 resulted in an accumulation of cells in the G2/M phase in a dose-dependent manner. Conversely, cells exhibiting low levels of DDB2 demonstrated an abrogation of the cell cycle arrest in the G2/M phase. The G2 arrest is initiated by the ATM-Chk2 signaling pathway, whereas its maintenance depends on ATR-Chk1 signaling [29, 30]. The ATM-Chk2 pathway is well established to be activated primarily in response to DSB [31]. The ATR-Chk1 pathway is typically stimulated by stress replication or UV light. However, Chk1 can also be activated in IR-induced DSB by the generation of structures containing single-stranded DNA coated with the replication protein A (RPA) complex, which forms near double-stranded DNA [32]. In our experiments, both pathways were activated by IR, yet only the cell cycle checkpoints Chk1 and Chk2 were differentially triggered in accordance with the level of DDB2 expression. DDB2 facilitated the activation of these checkpoints by phosphorylation, which was consistent with our prior observation of cell accumulation in the G2 phase. Upon activation, both Chk1 and Chk2 proteins can phosphorylate and promote the inactivation and degradation of CDC25A and CDC25C phosphatases, thereby preventing their inhibitory effects on the cyclin-dependent kinases CDK1 and CDK2, respectively, which ultimately allows cell cycle arrest [33, 34]. Given that the inhibition of CDC25C is responsible for blocking the CDK1-cyclin B complex, which initiates G2 arrest, we can therefore hypothesize that DDB2 may indirectly participate in blocking this complex by promoting Chk1 and Chk2 phosphorylation.

Our findings are consistent with prior research, which demonstrated an IR-resistant effect of DDB2 in NSCLC cells, promoting survival and inhibiting apoptosis following IR. The authors reported that DDB2 facilitated Chk1 phosphorylation, prolonged cell cycle arrest in the G2/M phase, and promoted DSB repair through the HR pathway. Interestingly, the study did not identify any impact of DDB2 on Chk2 activation, which contrasts with our findings in PDAC cells. Zou and colleagues were the first to describe the involvement of DDB2 in IR-induced DSB repair via the HR pathway. In their study, the authors postulated that DDB2 may enhance HR by promoting Chk1 activity, which, in turn, positively regulates HR repair through RAD51 phosphorylation [35]. In addition, the ATM-Chk2 signaling pathway can also be involved in HR by phosphorylating BRCA1 [36]. Although the efficacy of the HR pathway after IR was not examined, our NGS results demonstrated that all cell models were HRP. We hypothesized that DDB2 may enhance DSB repair through the HR pathway by phosphorylating Chk1 and Chk2 proteins in response to IR, as evidenced by the observed reduction of γH2AX foci. Conversely, we postulated that a low DDB2 expression may mimic a deficiency in the HR pathway, thereby conferring radiosensitivity to PDAC cells. Nevertheless, it cannot be excluded that DDB2 may also play a role in the NHEJ pathway. Further experiments are required to investigate this possibility.

A defect in the HR repair pathway can result in the sensitization of cells to PARPi, such as olaparib. PARP inhibition prevents SSB and leads to the formation of DSB that cannot be repaired in cells with HR repair defects. These DNA damages are handled by the NHEJ pathway, which is more prone to error, thus increasing genomic instability and ultimately leading to cell death [37]. In combination with RT, olaparib exhibited radiosensitizing properties regardless of the HR status by preventing PARP1-requiring repair and inducing DSB generation. In particular, olaparib has been demonstrated to exhibit dose-dependent radiosensitizing effects in several PDAC cell lines, including MIA PaCa-2, AsPC-1, PANC-1, and BxPC-3 [38–40]. In our cell models, we observed that the levels of DDB2 modulated the radiosensitizing effect of olaparib. Indeed, the induction of cell death following RT with olaparib pretreatment was markedly enhanced in T3M4 DDB2-low cells, whereas no significant increase in cell death was observed in Capan-2 DDB2-high cells. The concentrations of olaparib applied in our cell models (15 and 50 μM), were higher than those commonly utilized in the treatment of HRD cells (10–300 nM), yet remained clinically relevant. Indeed, the current recommended posology of olaparib (400 mg twice a day) used as a PARP inhibitor has been reported to result in a maximum plasma concentration varying between 2380 and 16,900 ng/mL (equivalent to a range of 4.5 to 38.9 μM) [41]. Additionally, a previous study has indicated that a higher concentration of olaparib is necessary to achieve radiosensitization, particularly in the context of HRP cells, like ours [11]. In consideration of the aforementioned information, it can be postulated that the concentrations utilized as radiosensitizers in our cells may have clinical relevance. Nevertheless, the optimal concentration for inducing a radiosensitizing effect in a clinical setting remains to be elucidated.

Previous studies have investigated the radiosensitizing effect of olaparib in combination with other agents. In their study, Vance et al., combined olaparib with a Chk1 inhibitor (AZD7762) and observed that Chk1 inhibition abrogated the G2 checkpoint and altered the HR repair pathway, while olaparib increased the accumulation of unrepaired DNA damage. The combination of PARP1 and Chk1 inhibitors achieved significantly higher radiosensitization of PDAC cells than monotherapy [38]. Another study observed similar mechanisms when olaparib was combined with AZD1775, an inhibitor of the cell cycle regulatory protein Wee1 [39]. These reports collectively suggest that alteration of the HR pathway and/or abrogation of the G2 checkpoint enhances the efficacy of olaparib. However, our results showed that DDB2 expression is linked to the G2 checkpoint and potentially associated with the HR pathway in our cell models. Consequently, we postulated that modulation of the radiosensitizing property of olaparib may be partly modulated through these mechanisms. It is noteworthy that we observed elevated Chk1 expression and activation in Capan-2 DDB2-high cells, which exhibited no radiosensitizing effect of olaparib. Given that Chk1 can promote cell cycle arrest and HR pathway, it can be speculated that increased Chk1 activity may reduce the efficacy of olaparib.

Although the modulation of radiosensitization by olaparib can be attributed to the IR-resistant effect of DDB2, we further investigated its impact on PARP1, which is the primary target of olaparib. Our results indicated that DDB2 enhanced the expression and activity of PARP1, resulting in a robust and quick increase in the level of PARylated proteins in cells exhibiting high DDB2 expression. In contrast, T3M4 DDB2-low cells exhibited a drastically reduced PARylated proteins level. To the best of our knowledge, the interaction between DDB2 and PARP1 has only been described in the NER pathway. In this context the two proteins collaborate closely together near the lesion site to enhance DNA repair. DDB2 enhances the catalytic activity of PARP1, while PARP1 activation promotes the PARylation of DDB2, thereby enhancing its stability and retention on chromatin through the prevention of its ubiquitination-mediated degradation [42–44]. Subsequently, histone PARylation by PARP1, stimulated by DDB2, results in the recruitment of the ALC1 helicase, facilitating chromatin remodeling. This is followed by the recruitment of XPC to activate the NER pathway [45]. It can be postulated that DDB2 may interact with PARP1 in the context of IR, in a manner analogous to that observed in the NER pathway. Furthermore, PARP1 is implicated in the HR pathway through the facilitation of the recruitment of MRE11, BRCA1, and RAD51 proteins, and in the NHEJ pathway by the stimulation of DNA-PK activity [46]. Consequently, it is also conceivable that DDB2 may promote the HR and/or the NHEJ pathways by stimulating PARP1 expression and activity.

The combination of a DNA damage response inhibitor with RT appears to be a promising therapeutic strategy. Following the demonstration of its efficacy as a radiosensitizer in pancreatic cancer cells, both in vitro and in vivo [47], the PARPi veliparib exhibited good tolerability and safety in a phase I study of locally advanced pancreatic cancer with gemcitabine and RT. Although no correlation was observed between OS and mutations in DNA damage response pathways, the authors reported that increased PARP3 expression and reduced RBX1 (ring-box protein 1) expression enhanced the sensitivity to PARP inhibition and improved OS [12]. In contrast, our findings indicate that reduced PARP expression is associated with a more favorable response. Nevertheless, our findings regarding the reduction of DDB2 expression in olaparib sensitization are in accordance with the aforementioned observations regarding the effect of decreased RBX1 expression on PARPi sensitivity improvement. Indeed, RBX1 forms the Cullin-RING ubiquitin ligase (CRL4) complex with CUL4A. Subsequently, this complex combines with the DDB1 and DDB2 proteins to form the larger CRL4^DDB2^ complex, which is involved in the NER pathway [48]. We therefore postulated that altering the NER pathway through the expression of DDB2 and RBX1 may increase cell sensitivity to PARP inhibition.

We previously reported that DDB2 sensitizes PDAC cells to chemotherapy. However, its expression is significantly decreased in PDAC patients, limiting its effect [24]. Conversely, low DDB2 expression may be beneficial in enhancing RT response for those patients. Furthermore, the observation that olaparib-pretreated RT improves response in DDB2-low cell models suggests that olaparib indication may be extended as a radiosensitizing agent in DDB2-low PDAC patients. This may enhance the only 4–7% of PDAC patients who benefit from this targeted therapy. In contrast, DDB2 inhibition could be a potential avenue for patients with high DDB2 expression to receive olaparib in combination with RT. The use of olaparib as a radiosensitizer could thus enhance the limited role of RT in the management of PDAC patients.

In conclusion, the present study demonstrates that DDB2 enhances radiation resistance in PDAC cells by promoting phosphorylation of Chk1 and Chk2 proteins, elevating PARP expression and activity, which ultimately leads to improved DSB repair. In summary, our findings suggest that DDB2 may serve as a potential predictive biomarker for RT response in PDAC patients, either with or without olaparib. This patient stratification, which identifies those more likely to respond to the combination of olaparib and RT, may have the potential to expand the therapeutic options for olaparib by enabling its use as a radiosensitizer in a wider range of patients.

## Materials and methods

### Cell lines

The Capan-2 (RRID: CVCL_0026) PDAC cell line was obtained from the American Type Culture Collection (ATCC) (Manassas, VA, USA). The T3M4 (RRID: CVCL_4056) PDAC cell line was kindly donated by Professor Jens Werner from the University of Heidelberg, Germany. T3M4 cells were transfected with a DDB2 CRISPR/Cas9 KO plasmid “T3M4 DDB2-low” or with a control CRISPR/Cas9 plasmid “T3M4 CTRL” and Capan-2 cells were transfected with a DDB2 CRISPR Activation plasmid “Capan-2 DDB2-high” or with a control CRISPR activation plasmid “Capan-2 CTRL” as previously described [24]. The cell lines were cultured in a humidified incubator with 5% CO_2_ at 37 °C in antibiotic-free RPMI 1640 medium (Gibco, Carlsbad, CA, USA) supplemented with 10% heat-inactivated fetal bovine serum (FBS) and 2 mM l-glutamine (Sigma-Aldrich Corp., St. Louis, MO, USA). The cells were periodically tested for the presence of *Mycoplasma* contamination using the VenorH GeM Mycoplasma Detection Kit (Minerva Biolabs GmbH, Berlin, Germany). All experiments were performed within 3–10 passages following the thawing of the cells.

### Irradiation exposure

Cells were subjected to a unique ionizing radiation dose at 2 Gy or 8 Gy. All the irradiations were conducted on a 6-MeV X-ray linear accelerator (Clinac 2100, Varian, Palo Alto, USA) at the Department of Radiation Oncology of the Institut de Cancérologie de Lorraine (Vandoeuvre-lès-Nancy, France).

### Olaparib pretreatment

T3M4 and Capan-2 cells models were seeded in six-well plates at a density of 1 × 10^4^ and 1 × 10^5^ cell per well, respectively. The cells were maintained in a culture medium for 48 h and then exposed to olaparib (Sigma-Aldrich Corp.) at a concentration of 15 µM for T3M4 cells and 50 µM for Capan-2 cells. After 24 h, the cells were exposed to 2 Gy irradiation (RT OLA) or not (OLA).

### Homologous recombination deficiency (HRD) testing

A total of four sections of 10 µm thickness from formalin-fixed paraffin-embedded (FFPE) cytological blocks obtained from our cellular models were used for DNA extraction. The DNA extraction was performed using the AllPrep DNA/RNA FFPE kit (Qiagen, Hilden, Germany) according to the manufacturer’s recommendations. DNA was quantified using the Qubit dsDNA High Sensitivity Assay kit (Thermo Fisher Scientific, Inc. Waltham, MA, USA). The homologous recombination deficiency (HRD) was analyzed using the SOPHIA DDM^TM^ HRD Solution panel (SOPHIA Genetics, Saint-Sulpice, Switzerland). An input of 50 ng of DNA was required for manual library preparation. This assay leverages a targeted capture-based technology to detect somatic and germline mutations in 28 HR-specific genes (*AKT1, ATM, BARD1, BRCA1/2, BRIP1, CCNE1, CDK12, CHEK1/2, ESR1, FANCA/L/D2, FGFR1/2/3, MRE11, NBN, PALB2, PIK3CA, PPP2R2A, PTEN, RAD51B/C/D, RAD54L,* and *TP53*). Moreover, this solution aims to identify the molecular consequences of HRD through low-pass Whole Genome Sequencing to determine the Genomic Integrity Index. Sequencing was conducted on the Illumina NextSeq 500/550® (Illumina, San Diego, California, USA). Bioinformatics analyses were conducted using the SOPHIA DDM^TM^ software, version 5.10.43, and pipeline 5.5.85 (SOPHIA Genetics).

### Clonogenic assay

T3M4 cells were seeded in six-well plates under standard conditions to achieve a minimum of 80% confluence. Subsequently, the six-well plates were subjected to ionizing radiation doses of 2 Gy and 8 Gy. Cells were detached using trypsin and counted. A total of 500 cells were then seeded in six-well plates and incubated in a culture medium for 7 days. Then, the cells were fixed in 70% ethanol for 15 min and stained with 1% crystal violet (Sigma-Aldrich Corp.) for 15 min. The number of colonies was counted, and the surviving fraction was normalized to the number of colonies obtained in untreated cells.

### Determination of γ-H2AX formation

Approximately 5 × 10^4^ T3M4 and 10 × 10^4^ Capan-2 cells were incubated on coverslips for 48 h and 72 h, respectively. The cells were fixed at 10 min, 1 h, and 24 h post-irradiation with a 4% paraformaldehyde/2% sucrose solution for 15 min. Subsequently, the cells were treated with PBS containing 50 mmol/L NH4Cl for 10 min to prevent fixative-induced fluorescence due to paraformaldehyde. Then, the cells were permeabilized with PBS containing 2% Triton X-100 for 10 min. The coverslips were then incubated with the primary antibody, a mouse anti-γ-H2AX (cat# 05-636, Millipore, Burlington, MA, USA, dilution 1:400 in PBS containing 3% BSA), for 1 h at 37 °C. The cells were subsequently washed with PBS and incubated with an anti-mouse IgG-FITC secondary antibody (#cat F2012, Sigma-Aldrich Corp., dilution 1:100 in PBS) for 20 min at 37 °C in the dark. The coverslips were washed five times and mounted onto glass slides using Vectashield Antifade mounting media counterstained with DAPI (Vector Laboratories, Burlingame, USA). Images were acquired using an Olympus AX70 microscope (Olympus, Tokyo, Japan) with a 100x objective. All cells were excited at the same exposure time. The average of foci was estimated by counting the number of foci in 50 nuclei in each condition. The count was performed independently twice with two different experimenters.

### Cell cycle distribution

The cells were seeded in six-well plates under standard conditions to achieve a minimum of 80% confluence. Then the six-well plates were exposed to an IR dose of 2 Gy. Twenty-four hours after irradiation, the cells were trypsinized and centrifuged for 10 min at 200×*g*. Subsequently, the pellet was washed once in phosphate-buffered saline (PBS) and fixed in ice-cold absolute ethanol. After centrifugation of 5 min at 300×*g*, 1 mL of ice-cold 70% ethanol was added, and the fixed cells were stored at −20 °C. The pellet was resuspended and washed twice in 2 mL of PBS, and then left for 2 h at room temperature. Following centrifugation of 5 min at 400×*g*, the pellet was resuspended in 500 µL PBS containing 20 µg/mL of propidium iodide (BD Biosciences, Franklin Lakes, NJ, USA), 0.1% Triton X-100 and 200 µg/mL RNase A (Qiagen). The cells were incubated in the dark for 15 min prior to analysis. Cell cycle determination was conducted using a BD Accuri™ C6 flow cytometer (Becton Dickinson, San Jose, CA, USA), and the fluorescence of at least 50,000 cells was analyzed using FCS Express software^TM^.

### Quantitative reverse-transcription PCR

Total RNA was extracted using the RNeasy Mini Kit (Qiagen, Hilden) in accordance with the manufacturer’s instructions. RNA was quantified using Invitrogen^TM^ Qubit RNA HS Assay Kit (Thermo Fisher Scientific). cDNA synthesis was conducted using the iScript^TM^ cDNA Synthesis Kit (Bio-Rad, Hercules, CA, USA) with 1 μg total RNA. Quantitative PCR was performed in triplicate on the LighCycler® 480 (Roche, Basel, Switzerland) using the LightCycler® 480 SYBR Green I Master kit. The primers were purchased from Eurogentec (Seraing, Belgium), and the sequences are provided in Table 2. The results were normalized with endogenous β-Actin, using the standard ΔΔCt method.

**Table 2:** Primer sequences used for RT-qPCR.

Gene	Forward sequence	Reverse sequence
*β-Actin*	AGAGCTACGAGCTGCCTGAC	AGCACTGTGTTGGCGTACAG
*ATR*	TGGTTGGAGAATGCTGGCTGC	ACATCACCCTTGGACCAGAGCC
*ATM*	GTTGCCAAGGTAGCTCAGTCT	CTGGCTCCCCTATACTTCTGTAG
*CHEK1*	GGGCAAAAAGGCCCCGAGTC	AGACCTGTGCGGGGTTCTGG
*CHEK2*	AGACACCCGTGGCTTCAGGA	CCTCGGCTTCCCCTTCACGG
*PARP1*	TGATAGCAGCAAGGATCCCAT	CCGTGCCACAGCAATCTTCG

### Western immunoblotting

The cells were lysed using RIPA lysis buffer (Merck, Darmstadt, Germany), which contained 2 mM phenylmethylsulfonylfluoride (PMSF) (Sigma-Aldrich Corp). The lysates were then centrifuged at 15,000×*g* for 20 min at 4 °C, after which the supernatants were stored at −80 °C. Proteins were quantified using the DC^TM^ Protein Assay Kit (Bio-Rad). Equal amounts of protein (30 μg) were separated by either 10% SDS-PAGE or by 5–15% SDS-PAGE and subsequently electro-transferred to polyvinylidene difluoride membranes (Bio-Rad). Then, the membranes were blocked with a solution of 0.1% Tween 20/Tris Buffered Saline (TBS) containing 5% non-fat milk for 1 h at room temperature under agitation. The primary antibodies mouse anti-α-Tubulin (cat# sc-23948, Santa Cruz Biotechnology, 1:1000), mouse anti-Chk1 (cat# 2360S, Cell Signaling, Danvers, MA, USA, 1:1000), rabbit anti-pChk1 (cat# 2344S, Cell Signaling, 1:500), rabbit anti-Chk2 (cat# 6334S, Cell Signaling, 1:1000), rabbit anti-pChk2 (cat# 2197S, Cell Signaling, 1:1000), rabbit anti-ATM (cat# 2873S, Cell Signaling, 1:1000), rabbit anti-pATM (cat# 5883S, Cell Signaling, 1:500), rabbit anti-ATR (cat# 13934S, Cell Signaling, 1:1000), rabbit anti-pATR (cat# 2853S, Cell Signaling, 1:1000), mouse anti-PARP (#cat 556362, BD Biosciences, 1:1000), rabbit anti-cleaved PARP (#cat 5625S, Cell Signaling, 1:1000), and rabbit anti-Poly/mono-ADP Ribose (cat#83732S, Cell Signaling, 1:1000) were incubated in blocking solution overnight at 4 °C. Secondary goat anti-rabbit HRP-linked (cat# 7074S, Cell Signaling, 1:2000) and horse anti-mouse HRP-linked (cat# 7076S, Cell Signaling, 1:2000) antibodies were applied for one hour at room temperature. The targeted proteins were then detected using the Clarity Western ECL kit (Bio-Rad) and visualized with the Azure C600 Camera (Azure Bio-systems, Dublin, CA, USA). The full-length western blots are available in the supplementary material.

### Crystal violet cell viability assay

Approximately 5 × 10^3^ T3M4 and 2 × 10^4^ Capan-2 cells were seeded in each well of a 96-well plate. After 48 h of incubation, the culture medium was replaced with a dilution range of olaparib (Thermo Fisher Scientific), and the cells were maintained in culture for a further 48 h. Subsequently, the cells were fixed with 70% ethanol for 10 min, followed by a 15-min incubation with 0.2% crystal violet in 20% ethanol. Following three washes, 0.1% acetic acid in 50% ethanol was added to each well, and the optical density was measured at 540 nm using a microplate reader (Multiskan As-cent; Thermo Fisher Scientific). The half-maximal inhibitory concentration (IC50) values were determined by a nonlinear regression using GraphPad Prism 9® (GraphPad Software, La Jolla, CA, USA). All experiments were conducted in triplicate on three independent assays.

### Apoptosis detection assay

T3M4 and Capan-2 cells were seeded in a six-well plate at a density of 1 × 10^4^ and 1 × 10^5^ cells per well, respectively. The cells were irradiated with or without olaparib pretreatment, as previously described. After 24 h, the cells were stained with annexin V/propidium iodide (BD Biosciences) according to the manufacturer’s instructions. Apoptosis was quantified within one hour by flow cytometry using a BD Accuri™ C6 flow cytometer.

### Statistical analyses

All experiments were conducted in triplicate, with three independent tests performed for each experiment. Statistical analyses were performed with GraphPad Prism 9® software, employing either a Student’s unpaired *t*-test or one-way ANOVA. The threshold for statistical significance was set at a *p* value ≤0.05.

## Supplementary information

Original western blots

Supplementary Table I and Supplementary Figure 1

## Data Availability

All data generated or analyzed during this study are included in this published article and its supplementary information files.
