# Supplementary material for: DDB2 expression lights the way for precision radiotherapy response in PDAC cells, with or without olaparib
Source: Cell Death Discov. 2024 Sep 27;10:411. doi: 10.1038/s41420-024-02188-9 (PMC11436999; doi:10.1038/s41420-024-02188-9)
Supplement: Supplementary file 1 — Original western blots [file 41420_2024_2188_MOESM1_ESM.docx]

**WESTERN BLOT MEMBRANE 1** – T3M4 WT, T3M4 CTRL, T3M4 DDB2-low (Antibodies: Chk1, pChk1, Chk2, pChk2, tubulin)


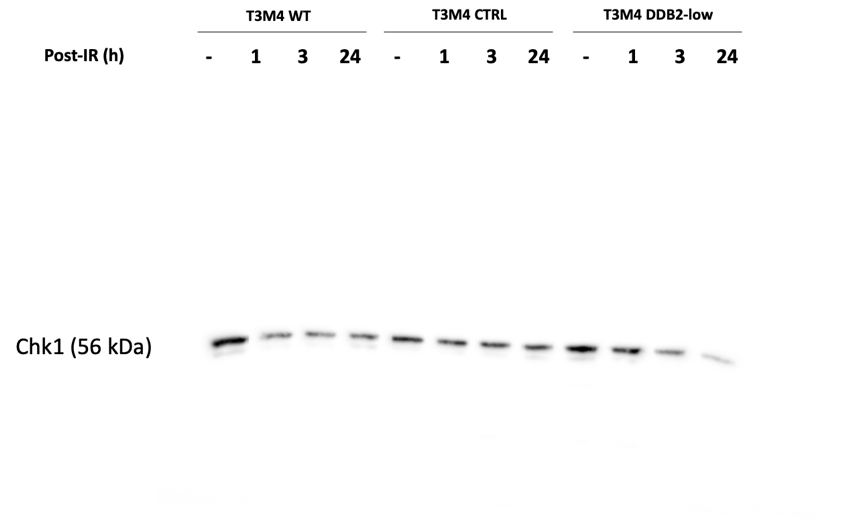

**WESTERN BLOT MEMBRANE 2** – T3M4 WT, T3M4 CTRL, T3M4 DDB2-low (Antibodies: ATR, pATR, ATM, pATM, tubulin)

**WESTERN BLOT MEMBRANE 3** – Capan-2 WT, Capan-2 CTRL, Capan-2 DDB2-high (Antibodies: Chk1, pChk1, Chk2, pChk2, tubulin)

**WESTERN BLOT MEMBRANE 4** – Capan-2 WT, Capan-2 CTRL, Capan-2 DDB2-high (Antibodies: ATR, pATR, ATM, pATM, tubulin)

**WESTERN BLOT MEMBRANE 5** – T3M4 CTRL (Antibodies: PARP1, cPARP, PARylated protein, tubulin)

**WESTERN BLOT MEMBRANE 6** – T3M4 DDB2-low (Antibodies: PARP1, cPARP, PARylated protein, tubulin)

**WESTERN BLOT MEMBRANE 7** – Capan-2 CTRL (Antibodies: PARP1, cPARP, PARylated protein, tubulin)

**WESTERN BLOT MEMBRANE 8** – Capan-2 DDB2-high (Antibodies: PARP1, cPARP, PARylated protein, tubulin)

**WESTERN BLOT MEMBRANE 9** – T3M4 CTRL & T3M4 DDB2-low (Antibodies: PARylated protein, tubulin)

**WESTERN BLOT MEMBRANE 10** – Capan-2 CTRL & Capan-2 DDB2-high (Antibodies: PARylated protein, tubulin)
