# Supplementary material for: DDB2 expression lights the way for precision radiotherapy response in PDAC cells, with or without olaparib
Source: Cell Death Discov. 2024 Sep 27;10:411. doi: 10.1038/s41420-024-02188-9 (PMC11436999; doi:10.1038/s41420-024-02188-9)
Supplement: Supplementary file 2 — Supplementary Table I and Supplementary Figure 1 [file 41420_2024_2188_MOESM2_ESM.docx]

Supplementary table I: Genomic instability index determined for cell models.

|  | **T3M4 CTRL** | **T3M4 DDB2-low** | **Capan-2 CTRL** | **Capan-2 DDB2-high** |
| --- | --- | --- | --- | --- |
| **Genomic instability index** | **-4.4** | **-3.2** | **-10.9** | **-11.1** |

Supplementary figure 1: DDB2 and PARylated protein expression levels.

PARylated protein expression were studied by western blot after 1 hour exposure to 2 Gy IR with or without olaparib pretreatment. Olaparib was administered 24 hours before. α-tubulin was used as a loading control.
